# AGE DIFFERENCES IN DISTINCT TYPES OF SOCIAL INTERACTIONS AND EMOTIONAL WELL-BEING

**DOI:** 10.1093/geroni/igac059.2897

**Published:** 2022-12-20

**Authors:** Yuxuan Chen, Kevin Chi, Laura Carstensen

**Affiliations:** Stanford University, Stanford, California, United States; Stanford University, Palo Alto, California, United States; Stanford University, Palo Alto, California, United States

## Abstract

Social interaction is central to emotional well-being. However, less is known about how the surroundings of social interactions are associated with emotional well-being and whether they differ by age. The present study examined age differences in the predication of emotional well-being as a function of two aspects of social interactions: (1) the extent to which individuals would rather be interacting with someone else and (2) the degree of closeness to the interactant(s). A sample of 190 people, aged 18 to 94 years, participated in an experience sampling study where they reported emotional experience and aspects of the social interaction at five randomly selected times each day across a one-week period for a total of 35 randomly selected occasions. Across age groups, the greater the extent to which people would rather be interacting with someone else was associated with higher levels of negative emotion and lower levels of positive emotion. However, these associations were more pronounced for younger adults, who compared to older adults, experienced greater increases in negative emotions and greater decreases in positive emotion in these contexts. Furthermore, younger but not older adults experienced lower levels of negative emotion when interacting with someone they knew well. Results highlight the important role that age plays in the link between daily social interactions and emotional well-being. Age differences in social interactions, emotional resilience, and situation selection will be discussed.

